# Editorial: Gary Felsenfeld (1929–2024)

**DOI:** 10.1093/nar/gkae503

**Published:** 2024-06-18

**Authors:** Thoru Pederson

**Affiliations:** Department of Biochemistry and Molecular Biotechnology, University of Massachusetts Chan Medical School, Worcester, MA 01605, USA


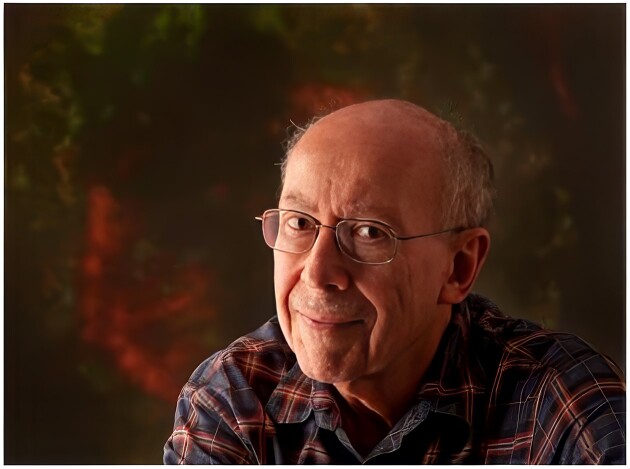


**Gary Felsenfeld**. Provided with permission by the U.S. National Institutes of Health Intramural Research Program.

On 1 May 2024, we lost a very impactful figure in our field- Gary Felsenfeld. He was 94 and still actively publishing, typical of the tremendous intellectual and scientific energy that characterized his entire career.

Gary graduated from Harvard College in 1951, having done an undergraduate thesis project with John Edsall, resulting in his first publication (1). He then undertook graduate work at Caltech in the laboratory of Linus Pauling (2), followed by a postdoctoral position at the University of Oxford with Charles Coulson. He subsequently joined the Laboratory of Neuroscience at the U.S. National Institute of Mental Health, soon rising to the position of Senior Assistant Scientist. In 1958–1961, he was appointed an Assistant Professor of Biophysics at the University of Pittsburgh and then joined the National Institute of Arthritis and Metabolic Diseases at NIH as Chief of the Section on Physical Chemistry. In 1997, he was appointed Chief of the Laboratory of Physical Chemistry at the renamed National Institute of Diabetes, Digestive and Kidney Diseases, continuing to also hold his other Section Chief position.

Gary's initial work at the NIH was on RNA homopolymers, culminating in the demonstration that poly(U) and poly(A) can form a triple-stranded structure (3). This was based on the observation that the 254 nm absorption reached a minimum not when the poly(U) and poly(A) were mixed at a molar ratio of 1:1, but rather when it was 2:1. When presenting this finding at meetings at the time, and in later career reflections, he always emphasized that it was his NIH colleague David Davies who helped him see what it might mean. As with the double helix, where Watson and Crick plausibly speculated on the base-pairing but did not actually identify it, the poly(U):poly(A) triple-stranded molecule suggested plausible pairing but the primary importance of the discovery was to establish an awareness the versatility of nucleic acid associations.

Gary and his colleagues later were among the first to investigate DNA folding including prescient studies of the value of micrococcal nuclease as a probe (4), foreshadowing its extensive applications to chromatin by his group and others. At this time, he also published, with his colleague Martin Gellert and others, an incisive study of how actinomycin binds to DNA (5), one that like so much of Gary's work was prescient and had many key ramifications. In another study that was timely, he determined the conformation of poly(U), a contribution that was very on point to the emerging question of RNA intramolecular secondary structure (6).

In due course, Gary turned his group's focus to chromatin. The histones had been long well defined (indeed one of them was among the first proteins ever completely sequenced, in a plant and animal with stunning evolutionary conservation). He was one of the first to recognize that the accessibility of chromatin to nucleases could be used to understand the disposition of histones on the DNA. With Robert Clark, Gary demonstrated that chromatin has periodic spacings of open versus nuclease-protected regions (7). This prescient finding was soon followed by related advances (8–11) that ushered in the nucleosome era. This was the period of Gary's career when I had the good fortune to first interact with him, when he took keen interest in my related work when I was just starting out (12). As a relevant point for this Editorial, it was soon after this time that Gary published extensively in *Nucleic Acids Research*, including forward-looking studies of histone heterotypic interactions on DNA (13–15) and on the high mobility group chromatin proteins (16,17). Chromatin continued to engage Gary as a biophysical and molecular entity and then, in due course, he and his laboratory logically and increasingly turned their attention to how chromatin structure relates to gene regulation. His group's meticulous dissection of the structure and regulatory network impinging on the chicken β-globin gene and other globin genes stands as a major achievement in the early era of the genomics field (18) and was one of the foundations for the subsequent success of the ENCODE project. Among many other key contributions at this time, his laboratory defined the ‘insulator’ protein CTCF that dictates the boundaries of DNA looping and expression (19,20).

Through his 70′s and 80′s Gary continued to move ahead in his field, with no slowing down whatsoever. At the time of his death, he had just published a major article (21), and with at least one more manuscript still under review.

No account of Gary Felsenfeld can omit his impactful mentorship, as was so manifest in a recent celebration of his career at which every former lab member attending spoke so movingly (22). We often enjoy such toasts but at the cited occasion these remarks were very moving and reflected a spectrum of relationships including an administrative assistant, a lab technician and several post-docs including a Nobel laureate, all of whom delivered the same message irrespective of their roles in the Felsenfeld lab: he was caring, thoughtful and special.

Gary leaves his beloved wife Naomi, three children, eight grandchildren and a great-grandchild. He also leaves a legion of colleagues worldwide who so greatly admired both his science and him, neither of which shall be forgotten.
